# Monthly Record of Deaths in the City of Buffalo

**Published:** 1857-05

**Authors:** C. L. Dayton

**Affiliations:** Health Physician


ART. III.—*Report of Mortality in Buffalo for the month of March*, 1857. By C. L. Dayton, M. D., Health Physician.

| DISEASES. | No. | Males. | Females. |
|---|---|---|---|
| Asthma, | 1 | | 1 |
| Asphyxia Immersorum, | 1 | 1 | |
| Accident, | 1 | 1 | |
| Amenorrhœa, | 1 | | 1 |
| Abscess Hepatic, | 1 | 1 | |
| Apoplexia, | 5 | 4 | 1 |
| " Pulmonun, | 1 | 1 | |
| Chorea, " | 1 | | 1 |
| Cholera Infantum, | 2 | 1 | 1 |
| Cephalitis, | 3 | 1 | 1 |
| Croup, | 8 | 6 | 2 |
| Dysentery | 2 | 2 | |
| Diarrhœa, | 3 | 3 | |
| Debilitas Senilis, | 6 | 2 | 4 |
| Dentitio, | 4 | 2 | 2 |
| Eclampsia, | 22 | 15 | 7 |
| Epilepsy, | 1 | | 1 |
| Enteritis, | 2 | | 1 |
| Febris Typhoid, | 5 | 3 | 2 |
| " Scarlatina, | 38 | 21 | 17 |
| " Scarlatina sequelæ of, | 6 | 3 | 3 |
| " Ignotus, | 1 | | 1 |
| Favus, | 1 | 1 | |
| Gastritis, | 3 | 2 | 1 |
| Hæmorrhage Post Partum, | 1 | | 1 |
| Hydrocephalus, | 7 | 3 | 3 |
| Hyperæmia Cerebralis, | 1 | 1 | |
| " Pulmonalis, | 1 | | 1 |

### REGISTER OF MORTALITY—Continued.

| DISEASES. | No. | Males. | Females. |
|---|---|---|---|
| Hydrops, | 1 | 1 | |
| Ignotus, | 34 | 17 | 12 |
| Intemperance, | 1 | 1 | |
| Laryngeal Ulcer, | 2 | 1 | |
| Myelitis, | 1 | 2 | |
| Morbus Brightii, | 2 | 1 | 1 |
| " Cordis, | 5 | 2 | 3 |
| " Hepatis, organic, | 2 | | 2 |
| Marasmus, | 3 | 2 | 1 |
| Pertussis, | 2 | 2 | |
| Parturitio, | 2 | | 2 |
| Pneumonitis, | 13 | 8 | 3 |
| Pleuro-dynia, | 1 | 1 | |
| Pyolæmia, | 1 | 1 | |
| Pleuritis, | 1 | | |
| Paralysis Pulmonalis, (!) | 1 | 1 | |
| Rheumatism, | 2 | 1 | 1 |
| Scrophula, | 2 | 2 | |
| Still-born, | 6 | 3 | 1 |
| Syphilis Congenital, | 1 | 1 | |
| Stomatitis Materna, | 1 | | 1 |
| Tumor Abdominis, | 1 | | 1 |
| " Scalp, | 1 | 1 | |
| Tuberculosis Pulmonalis, | 21 | 12 | 9 |
| Total, | 235 | | |

### SEXES.

| | |
|---|---|
| Males, | 133 |
| Females, | 89 |
| Sex not given, | 13 |
| Total, | 235 |

### AGES.

| | |
|---|---|
| Still-born, | 6 |
| 1 day, | 5 |
| 1 day and 30 days, | 18 |
| Between 1 month and 6 months, | 30 |
| " 6 months and 12 " | 27 |
| " 1 year " 3 years, | 51 |
| " 3 " " 5 " | 15 |
| " 5 " " 10 " | 13 |
| " 10 " " 20 " | 7 |
| Total number under 10 years, | 165 |

| | |
|---|---|
| Between 20 years and 30 years, | 15 |
| " 30 " " 40 " | 23 |
| " 40 " " 50 " | 9 |
| " 50 " " 60 " | 4 |
| " 60 " " 70 " | 6 |
| " 70 " " 80 " | 3 |
| " 80 " " 90 " | 2 |
| " 90 " " 100 " | 0 |
| " 100 " | 1 |
| Total number over 10 years, | 70 |

Ages not given, 0 Total, 135

### NATIVITIES.

| | |
|---|---|
| American, (white,) | 143 |
| " (colored,) | 1 |
| German, | 38 |
| Irish, | 20 |
| English, | 5 |
| Canadian, | 3 |
| French, | 2 |
| Scotch, | 0 |
| Swiss, | 1 |
| Country not given, | 22 |
| Total, | 235 |

